# Label-free optical interferometric microscopy to characterize morphodynamics in living plants

**DOI:** 10.3389/fpls.2023.1156478

**Published:** 2023-05-22

**Authors:** Samira Ebrahimi, Guillermo Moreno-Pescador, Staffan Persson, Liselotte Jauffred, Poul Martin Bendix

**Affiliations:** ^1^ Copenhagen Plant Science Center, Department of Plant and Environmental Sciences, University of Copenhagen, Frederiksberg, Denmark; ^2^ Biocomplexity, Niels Bohr Institute, University of Copenhagen, Copenhagen, Denmark; ^3^ Joint International Research Laboratory of Metabolic and Developmental Sciences, State Key Laboratory of Hybrid Rice, School of Life Sciences and Biotechnology, Shanghai Jiao Tong University, Shanghai, China

**Keywords:** digital holographic cell imaging, optical coherence tomography, label-free microscopy, material transport, intereferometric imaging, plant cells and tissues, speckle imaging, plant morphodynamics

## Abstract

During the last century, fluorescence microscopy has played a pivotal role in a range of scientific discoveries. The success of fluorescence microscopy has prevailed despite several shortcomings like measurement time, photobleaching, temporal resolution, and specific sample preparation. To bypass these obstacles, label-free interferometric methods have been developed. Interferometry exploits the full wavefront information of laser light after interaction with biological material to yield interference patterns that contain information about structure and activity. Here, we review recent studies in interferometric imaging of plant cells and tissues, using techniques such as biospeckle imaging, optical coherence tomography, and digital holography. These methods enable quantification of cell morphology and dynamic intracellular measurements over extended periods of time. Recent investigations have showcased the potential of interferometric techniques for precise identification of seed viability and germination, plant diseases, plant growth and cell texture, intracellular activity and cytoplasmic transport. We envision that further developments of these label-free approaches, will allow for high-resolution, dynamic imaging of plants and their organelles, ranging in scales from sub-cellular to tissue and from milliseconds to hours.

## 1 Introduction

Advancement of imaging technology is essential to gain a deeper understanding of structural and molecular organization in plants. A variety of different marker-based imaging approaches, i.e. fluorescence microscopy, have been developed to image the dynamical structures of living plant cells, relying on dyes and different contrast agents (Ovečka et al., 2018; Pain et al., 2019; Bayle et al., 2021; DeVree et al., 2021; Ovečka et al., 2021). The unique strength of fluorescence techniques lies in the specific markers, allowing identification of sub-cellular structures and molecular transport of organelles between different cellular compartments (Aniento et al., 2021). Long-term imaging of plant tissues or individual cells is of particular interest, enabling analyses of intra-cellular dynamics of free vesicular diffusion or active transport by molecular motors’ interaction with the cytoskeleton (Kang et al., 2021). Additionally, fluorescence imaging enables detection of chemical events like pH changes (Resentini et al., 2021) and calcium signaling (Gjetting et al., 2013) which is hard to achieve by other imaging modalities. Thus, a precise understanding of plant activity may assist in optimizing plant growth with consequences for e.g. sustainable agriculture and food production.

Although many efforts have been made to elucidate plant cell dynamics, we still lack suitable methods for long-term quantitative visualizations of cell-growth, functionality, and how these relate to sub-cellular transport. For this purpose, fluorescence microscopy is essentially limited by the lack of photostable fluorescent marker proteins, phototoxicity and the high complexity of membrane compartments and their trafficking pathways.

By contrast, label-free optical interferometry is a non-contact method to measure the structural dynamics of the cells in its native environment without the requirement of markers (Zernike, 1955; Gutiérrez-Medina, 2022). Thus, it does not require any specific sample preparation, so there is no balancing of phototoxicity from contrast agents with specificity (Curl et al., 2004). Instead, this imaging method is based on contrast enhancements, due to the phase changes of the light as a result of the relative refractive index (RI) difference between the structure of interest and the environment (Dyson and Allibone, 1950).

Here, we review some recent optical coherent interferometric methods with potential application in plant research, such as biospeckle imaging and interferometry (Zdunek et al., 2014; De la Torre et al., 2016; Pandiselvam et al., 2020), optical coherence tomography (Schmitt, 1999), and digital holographic microscopy (Kim, 2010; Ebrahimi et al., 2018). Given the high sensitivity of interferometric methods and high acquisition rates of hundreds to thousands of frames per second, these methods are particularly suitable to monitor fast processes in plants from macroscopic tissue imaging to sub-microscopic scales of cells and organelles over long time-scales.

## 2 Biospeckle laser imaging

**Figure 1:**
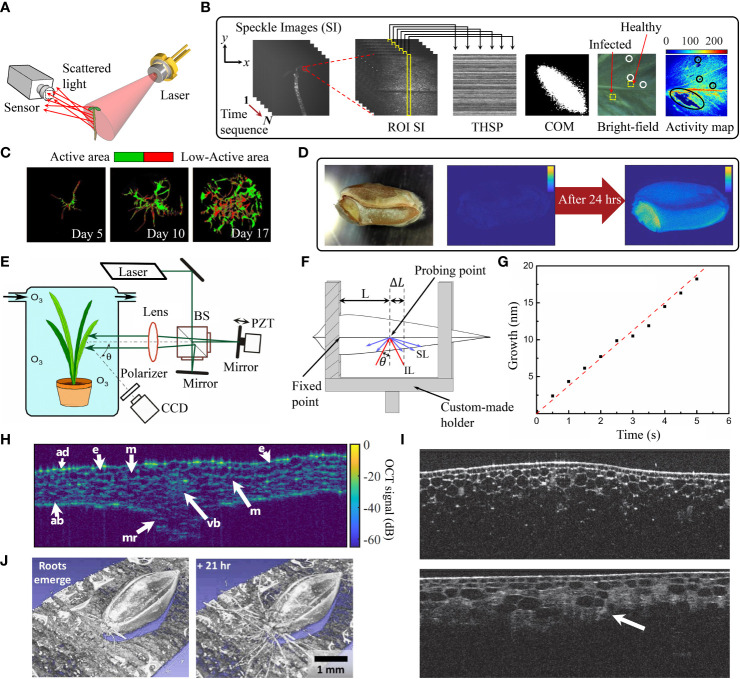
**(A)** Schematic setup of biospeckle imaging. **(B)** Construction of THSP matrix, an example of COM and bioactivity map [adapted from Ansari et al. (2018)]. **(C)** Biospeckle activity of hairy roots after different cultivation times (Schott et al., 2020). **(D)** Germination of a wheat seed. Left image: Bright-field image, right images: Bioactivity maps at 0 hrs and 24 hrs after germination (Sutton and Punja, 2017). **(E)** Left plot: Schematic setup of statistical interferometry technique based on Michelson interferometer. PBS, Polarization beam-splitter; PZT, Piezoelectric transducer [adapted from Kadono and Kobayashi (2009)], **(F)** Optical path changes as a result of leaf elongation. IL: Illumination light and SL: Scattered light from the leaf (Silva et al., 2016) **(G)** Elongation of a Chinese chive leaf (Kadono and Kobayashi, 2009). **(H)** Cross-section image of an Arabidopsis leaf, acquired by OCT technique reprinted with permission from (de Wit et al., 2020 © The Optical Society). e: Epidermal cell, m: Mesophyll cell, vb: Vascular bundle, mr: Midrib, ad: Adaxial side, ab: Abaxial side. **(I)** OCT histological images of onion for normal onion tissue (top plot) and onion with bruise defects (highlighted by white arrow in bottom plot) (Meglinski et al., 2010). **(J)** 3D view of OCT images of root growth in a switchgrass seed a few minutes after emergence of roots (left) and after 21 hrs (right) (reprinted with permission from Larimer et al., 2020 © The Optical Society).


Speckles arise when a coherent light source transmits through a medium with heterogeneous internal structure or is back-scattered from a rough surface. Due to the interference of light, a pattern of bright and dark voxels appears at the observation plane (Goodman, 2020). When imaging biological tissues, biospeckles, will be moving in space and time. Specifically, any chemical, physical and physiological processes, like organelle movement, cytoplasmic streaming and cell division will cause redistributions of the biospeckle pattern and intensity distribution. Thus, this method can be accurately used for reporting dynamics using temporal correlation of irradiance changes. The conventional biospeckle imaging configuration requires illumination of the sample using divergent light from a coherent laser source and the resulting scattered light is collected by a high speed CCD or CMOS sensor (**Figure 1A**). Multiple numerical and analytical methods can be applied for analysis of the recorded biospeckle patterns. A numerical method for bioactivity measurement is based on computation of the co-occurrence matrix (COM) of the time history speckle patterns (THSP) for a region of interest (ROI) within the sample (Oulamara et al., 1989). The COM provides information on the temporal intensity differences. The elements in the COM represent how many times pixels exhibit a certain intensity difference in successive images, see Arizaga et al. (1999) for a mathematical description of the matrix. The elements are arranged in a square matrix with diagonal elements, representing constant intensity values whereas non-diagonal elements represent sample locations with changed intensity. Elements further away from the diagonal represent increasing temporal variability of the intensity. Hence the interpretation of the COM can be used to estimate the sample activity changes by quantifying the spread of non-zero elements around the principal diagonal of the matrix (see example of a COM in **Figure 1B**. Computation of the inertia moment (*IM*) of COM provides a quantification of activity changes in the image. Mathematically, *IM* is calculated from the sum of the multiplication of COM values by their squared distances from the principal diagonal (Arizaga et al., 1999),

(1) $$IM=\sum_{i,j}COM_{i,j}|i-j{|}^{2}.$$

according to this equation, a COM with only non-zero diagonal elements has IM=0 , corresponding to zero activity. Temporal variations in intensity lead to non-zero values away from the diagonal, leading to larger values of IM .

To measure spatial biospeckle activity, Fuji et al. proposed to calculate the sum of the differences between consecutive pixels from a series of *N* biospeckle distributions (Fujii et al., 1985):


(2) $$BA\left(x,y\right)=\sum_{n=1}^{N}\frac{\left|P_{n}\left(x,y\right)-P_{n+1}\left(x,y\right)\right|}{P_{n}\left(x,y\right)+P_{n+1}\left(x,y\right)},$$

where *n* is the image index for the sequence of n=1,2,…,N images and *P_n_* is the intensity value for each pixel with *x* and *y* coordinates. This equation highlights any temporal intensity variations in the biospeckle field. **Figure 1B** shows the speckle analysis including an example of THSP, COM and bioactivity map. In addition to the Fuji method, other algorithms have also been used for bioactivity assessment such as speckle contrast, generalized difference method (Arizaga et al., 2002), and temporal laser speckle contrast analysis (Cheng and Duong, 2007).

Recently, the biospeckle imaging method has been applied in neuroscience, dermatology and ophthalmology, e.g., monitoring blood flow in retina, skin and brain (Cheng and Duong, 2007; Boas and Dunn, 2010; Abd El-Ghaffar and Khalil, 2021). In plant and agricultural studies, biospeckle imaging has been used not only to monitor development, but also for detection of defects and diseases, for instance, to measure the quality of crops (Ribeiro et al., 2013; Sujayasree et al., 2022) and changes in maturation and ripening of vegetables and fruits (Romero et al., 2009; Arefi et al., 2016). It was observed that biospeckle activity was changed during the maturation process. Furthermore, biospeckle activity can provide a unique signature of leaves, which has been employed to assess the healthy and infected regions of a plant tissue, so that any local infection can be distinguished from the healthy regions by different (lower) bioactivity levels (Ansari and Nirala, 2015; Ansari et al., 2018) (see bright-field image and activity map in **Figure 1B**). In a recent study, the potential of biospeckle imaging has been shown in detection of endophytic colonization in leaves (D’Jonsiles et al., 2020). The method has also been used to image bioactivity in the root and apical hairy root tissues (Braga et al., 2009; Ribeiro et al., 2013; Schott et al., 2020; Schott et al., 2022). **Figure 1C** shows the biospeckle activity in heterogeneous hairy root culture in a cultivation assay over time. The root apex has the highest biospeckle intensity, because of fast cell division. However, when exposed to mechanical stress (e.g., thigmo-stimulation), biospeckle intensity decreases which may reflect depolymerization of actin. But more research is needed by correlating these interferometry data with microscopy of fluorescent actin.

Another application of biospeckle imaging in plants is the seed germination test prior to seed selection. Early diagnosis and treatment of seeds against pathogens minimizes the requirement for chemical treatments in mature plants. It is, thus, important for the quality of agricultural products (Vivas et al., 2017). **Figure 1D** shows, from left to the right, the bright-field image of a seed and the bioactivity map at 0 hrs and after 24 hrs of germination (Sutton and Punja, 2017). Recently, biospeckle techniques were used to monitor: the germination of maize seeds, contaminated with fungi and after treatment with a bioprotector (Silva et al., 2018), the effect of different priming treatments on seed germination (Singh et al., 2020), the effect of temperature and initial moisture content during soybean seed germination (Thakur et al., 2022) and sprouting damage in wheat seeds (Sutton and Punja, 2017). A strong correlation between the biospeckle activity and germination percentage was seen and the results were in a good agreement with standard germination tests. As a further application of biospeckle imaging, (O’Callaghan et al., 2018) automatically selected living nematodes in soil using biospeckle selective plane illumination microscopy for studying the plant-nematode interactions in the rhizosphere.

## 3 Statistical speckle interferometric techniques

Statistical interferometric methods have emerged for mapping of rough surface optical properties such as deformation as the primary applications (Kadono and Toyooka, 1991; Kadono et al., 2001). The principle behind statistical interferomentry is based on the interference of two fully developed speckle fields instead of the traditional approach of superimposing a reference beam reflected from an accurate flat mirror, with a beam modulated by the sample. In fully developed speckle fields, the phase of speckles is randomly distributed between −π and π , resulting in a probability density function (PDF), that is uniformly distributed. This feature is used to measure the object’s phase. The stability of the speckle field statistics is important for a reliable measurement which is satisfied in most biological samples.

This method has provided continuous measurements of elongation of roots and entire seedlings (Rathnayake et al., 2007). The conventional optical setup for this investigation is an amplitude division interferometer, e.g., a Michelson-type interferometer (**Figure 1E**) (Wang L. et al., 2021), or a common-path interferometer (Muthumali DeSilva et al., 2017). The latter is based on division of light wavefront into two beams, illuminating the sample, and collecting the two superimposed biospeckle fields. Since any nano-scale changes in elongation causes a phase difference in the interference pattern, the elongation rate is given as the phase change through PDF of the biospeckle phase. To this end, the plant is fixed using a holder (see **Figure 1F**) and the in-plane displacement relative to the fixed point is measured: $\text{Δ}L=\frac{\lambda \Delta \varphi }{4\pi sin\;\theta }$ , where λ is the wavelength of the laser, θ is the angle between the illumination beam and the normal to the detection plane, and Δφ , represents the phase difference between the two interfering biospeckle fields. This method has been used for multiple applications in plants: instantaneous tracking of nano-scale growth fluctuations of leaves under stress (see **Figure 1G**) (Muthumali DeSilva et al., 2017), monitoring both the quality of pine seedlings through root elongation rate (Wang L. et al., 2021) and the short-term effects of exogenous plant hormones on rice (Kabir et al., 2020), studying the nano-scale growth behavior of fungi-infected (Rathnayake et al., 2008) and uninfected seedlings under ozone exposure at different concentrations (Rathnayake et al., 2007). The authors observed that fungal infection results in higher root growth.

## 4 Optical coherence tomography

Optical coherence tomography (OCT) provides cross-sectional images of the internal structure of biological tissues with axial resolution of 5−7 μ m (Kirby et al., 2022). For OCT, a light beam from a broadband light source is divided into a reference and a sample beam. The back-scattered light from the sample, interferes with the reference and the resulting pattern is used to calculate the reflectivity versus depth profile of the tissue samples *in vivo* (Podoleanu, 2012). The OCT technique enables digital quantitative phenotyping with high sensitivity in plant anatomy, plant disease identification, and qualification of the agricultural products (Li et al., 2022a, Li et al., 2022b). The method has a high potential for identification of seed viability during an early stage of germination and thereby facilitates seed selection (Lim et al., 2019; De Silva et al., 2022). Biospeckle OCT has been used to screen for internal structural changes of pea seeds during germination process (Li X. et al., 2022), the effect of Zn concentration on lentil seed germination and seedling growth (De Silva et al., 2021), detection of microstructural changes in leaves during senescence (Anna et al., 2019) and measurements of the RI and thickness of leaves (de Wit et al., 2020). **Figure 1H** shows the central cross-section of an infiltrated Arabidopsis leaf wherein cell layers, epidermal cells, mesophyll cells, as well as vascular bundles can be seen. OCT has additionally been utilized for non-invasive *in vivo* detection of internal defects and structures of onions. **Figures 1I** demonstrates the cross-sections of healthy onion sample (top image) and onion with bruise defects (bottom image) (Meglinski et al., 2010). Moreover, OCT can be used to study root growth in soil and understand the relationship between roots, nutrients, and pathogens (Larimer et al., 2020). The emergence of roots in switchgrass seed and the root growth after 21 hrs are presented in **Figure 1J**.

## 5 Digital holographic microscopy

**Figure 2:**
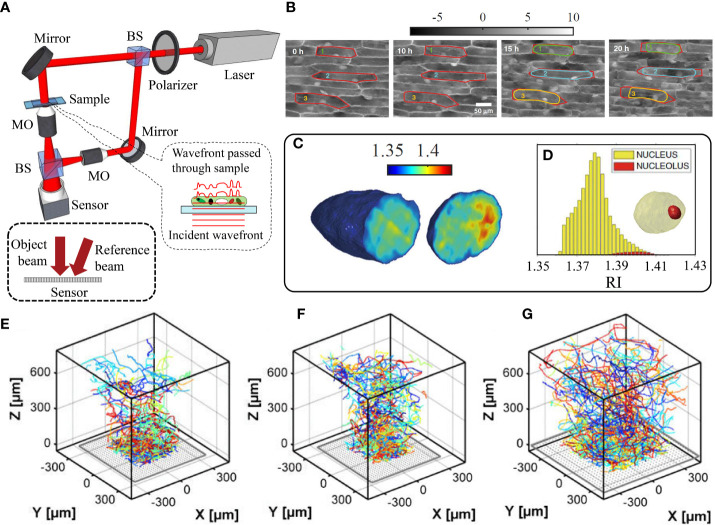
**(A)** Digital holographic microscopy setup based on Mach-Zehnder interferometry. BS: Beam splitter, MO: Microscope objective. **(B)** Contrast enhancement of phase maps through dehydration process over time. The cell walls are shown in red and the cell membranes are marked in green, cyan, and yellow. **(C)** 3D refractive index tomogram slices of a cell’s nucleus, **(D)** the histogram of the refractive index distribution of the nucleus and its nucleolus, depicted in the inset 3D model (Wang Z. et al., 2021). The 3D trajectories of zoospores of U. linza macroalga in the vicinity of glass coated by **(E)** polyethylene glycol, **(F)** acid, and **(G)** tridecafluoroctyl-triethoxysilane. Reprinted with permission from (Heydt et al., 2012).

Digital holographic microscopy (DHM) is a another label-free quantitative phase imaging approach, which builds on the use of the interference between a reference light beam and an object beam, employing an interferometer such as Mach-Zehnder configuration (Anand et al., 2010) (see **Figure 2A**). The technique can be used for quantitative phase imaging of micron-sized specimens through reconstruction of both the optical path length and intensity distributions (Kim, 2010; Ebrahimi and Dashtdar, 2021). Consequently, DHM has been used to measure biochemical, morphological, and mechanical properties of bio-samples (Dubois et al., 2006; Quan et al., 2018). Some recently proposed configurations of DHM are easily adaptable with other modalities, such as optical tweezers (Ebrahimi et al., 2014) and fluorescence microscopy (Kumar et al., 2020). The reconstruction process is based on numerically solving the diffraction integral, which allows for high precision numerical auto-focusing of moving samples for real-time 3D tracking, without any requirement for mechanical focus adjustments (Langehanenberg et al., 2011). Another application of DHM is holographic tomography by multiple-angle illumination, which yields the sample’s 3D RI distribution (Balasubramani et al., 2021). Given the orientation between reference and object beams, DHM can be achieved based on either on-axis geometry, where both object and reference beams propagate along the same path (Yamaguchi, 2006), or off-axis geometry, where a tilt is introduced between the two interfering beams, which enables single-shot imaging (Sánchez-Ortiga et al., 2014). Angular spectrum propagation (ASP) approach can be utilized for the numerical analysis of these off-axis holograms. ASP is based on the Fourier transform analysis and propagation of the object wavefront in Fourier space and quantitatively yields the sample’s thickness and RI. See Anand et al. (2010) for further mathematical description of ASP method.

DHM has proven to be a versatile technique for studying the dynamical processes and morphologies of various biological specimens, including red blood cells (O’Connor et al., 2021; Kumar M. et al., 2022), yeast cells cells (Rappaz et al., 2009), microorganisms (Lee et al., 2011), neurons (Marquet et al., 2013), sperm (Dubey et al., 2019), and cardiomyocytes (Jaferzadeh et al., 2020). Although the use of DHM in plant studies has been limited, Vora and Anand utilized the technique to observe changes in volume and texture of onion epidermal cells (Vora and Anand, 2014), demonstrating that the cells decrease in volume and become rougher over time, likely due to water loss. Jiang et al. also leveraged dual wavelength DHM to analyze intracellular activities, nucleus dynamics, cell transport, cell wall, and cytoskeleton compartments in onion’s epidermal cells (Huang et al., 2021). Ferraro et al. used DHM to study the dehydration process of plants and enhance the imaging of intracellular structures in living epidermal cells (Wang et al., 2019). The phase maps of the cells’ contraction (plasmolysis) over 20 hours, with plasma membranes (green, cyan, and yellow) separated from cell walls (red), are shown in **Figure 2B**. The nucleus tomograms and refractive index histograms for the nucleus and nucleolus are displayed in **Figures 2C, D** respectively. Furthermore, decreasing the water content of the plants leads to significant nucleus rotation as a result of turgor pressure reduction (Wang Z. et al., 2021). After acquiring the nuclei DHM tomograms, 3D refractive index, dry mass, 3D dynamics and biological volume were measured. Kumar V. et al. (2022) also used DHM to evaluate the viability of unstained pollen grains, a critical aspect of plant reproduction. Their results indicate that non-viable pollen samples have a significantly smaller phase than viable samples due to a reduction in cytoplasmic material. As a further application of DHM in plants, it is employed to follow the interactions between solid and liquid particles in the air (e.g., metals, acids, aerosols, and dust) and leaves (Go et al., 2021). The 3D detection of particles and distinction between freely diffusing and adhered particles was performed using an autofocusing algorithm, image segmentation and velocity analysis. In a similar study, DHM was utilized to track the 3D swimming zoospores of a green alga (*Ulva linza*) near a surface and understand the mechanism of surface exploration (Heydt et al., 2007; Heydt et al., 2012). The spores’ 3D trajectories over 10 min, for three different surface coatings, are shown in **Figures 2E–G**. Finally, DHM phase reconstruction was applied in amarine environment to identify and classify various micro-plastic fragments, which can pose a health risk to humans through the food chain (Merola et al., 2018).

## 6 Discussions and perspectives

Interferometric imaging contains a number of advantages for imaging plant cells. First, long-term imaging is readily performed without loss of signal and without the drawbacks associated with molecular labeling (Gjetting et al., 2013). Fluorescent imaging of plant tissue and organs containing chlorophyll, which causes autofluroescence and limits transparency, is very challenging. Secondly, interferometric methods provide an alternative imaging platform, which is still largely unexploited. In this review, a few state-of-the-art techniques for label-free interferometric imaging of living plants in their native environment have been presented, including biospeckle imaging and interferometry, optical coherence tomography and digital holographic microscopy. These interferometric techniques have the potential to provide us with morphological analysis, measurements of nanoscopic displacements and imaging of intracellular dynamics in plant and agricultural products. Examples of some of the pioneering studies include imaging of seed germination, defects and diseases, bioactivity, cytoplasmic streaming, cytoskeletal organization, environmental interactions and high-resolution imaging of plant growth. However, since interferometric imaging techniques suffer from lack of specificity, frequency decomposition approach may help to distinguish between different dynamical processes within the cells and tissues.

Integration of fluorescent channels with the compatible interferometric modalities may offer new ways to perform live imaging of plants with high specificity. We envision this nondestructive and label-free approach to be used for investigation of vesicle traffic, cell growth, cytoskeleton organization, membrane features, cell wall cellulose synthesis and pathogen-plant interactions in combination with quantitative multi-channel imaging approaches. Combining these two imaging methods additionally allows one to perform fluorescent based chemical imaging like calcium signaling Resentini et al. (2021), and pH detection Littlejohn et al. (2014) in conjunction with interferometric imaging thus allowing structural changes in the cytoplasm to be correlated with molecular and ionic concentrations. One novel example in which fluorescent imaging and interferometry are combined is Rotating Optical Coherent Scattering (ROCS) microscopy developed by the Rohrbach group (Jünger et al., 2016; Jünger et al., 2022). ROCS allows high speed imaging at sub diffraction lateral resolution ( ∼ 150 nm) and is suitable for imaging surface structures of plant tissues in combination with fluorescent imaging like total internal reflection fluorescent microscopy.

Finally, we emphasize the importance of using numerical approaches for accurate interpretation of the interferometric signals. Various methods already exist, which transform raw data into meaningful readout like distance, morphology or activity. Well-established algorithms based on particle tracking, like particle imaging velocimetry (Wereley and Meinhart, 2010; Baiyin et al., 2021), could also be adapted to quantify flow patterns in plant cells by isolating the translational component of interferometric signals. The list of applications of interferometry hereby seems endless and its potential in plant science should be realized in the near future.

## Author contributions

SE wrote the first draft and composed the figures. All authors critically reviewed and commented on the manuscript. All authors contributed to the article and approved the submitted version.
